# Internalized Stigma in Acne Vulgaris Patients and Relationship with Quality of Life, Disease Severity

**DOI:** 10.3390/healthcare13121359

**Published:** 2025-06-06

**Authors:** Nurperihan Tosun, Mustafa Tosun, Mahir Dığış, Mustafa Younis

**Affiliations:** 1 Department of Health Management, Faculty of Health Sciences, Sivas Cumhuriyet University, Sivas 58140, Türkiye; nurperihankarabulut@gmail.com; 2 Department of Dermatology, Faculty of Medicine, Sivas Cumhuriyet University, Sivas 58140, Türkiye; 3 Department of Dermatology, Faculty of Medicine, Fırat University, Elazığ 23200, Türkiye; mahirdigis33@gmail.com; 4 Department of Health Policy and Management, Jackson State University, Jackson, MS 39217, USA; j00103323@jsums.edu

**Keywords:** internalized stigma, acne vulgaris, quality of life, disease severity

## Abstract

**Background/Objectives**: Acne vulgaris (AV), a common dermatological condition in adolescence, has been widely recognized not only for its physical impact but also for its significant psychological and social consequences, particularly the internalization of stigma. This study specifically aimed to evaluate the state of internalized stigma in adolescents with AV and its relationship with quality of life and disease severity. Additionally, we sought to identify and assess the factors associated with internalized stigma. **Methods**: A total of 179 patients with AV were included in this cross-sectional observational study. We employed a convenience sampling strategy. The Internalized Stigma Scale (ISS) was used to assess patients’ stigma. The Acne Quality of Life Scale (AQLS) and the Dermatology Life Quality Index (DLQI) were used to assess patients’ quality of life. The Global Acne Grading System (GAGS) was used to assess disease severity. **Results**: Cronbach’s alpha coefficient for the ISS was determined to be 0.79. In our study, the mean total ISS scores for patients with AV were notably high. The ISS was significantly positively correlated with the AQLS score (*r* = 0.653, *p* < 0.001), DLQI score (*r* = 0.487, *p* < 0.001), and GAGS score (*r* = 0.257, *p* = 0.006). Linear regression analysis was performed to predict the ISS variable. Accordingly, the AQLS positively and significantly predicts (*β* = 0.521, *p* < 0.001). **Conclusions**: AVs often experience high levels of stigma. Internalized stigma is strongly associated with reduced quality of life and increased disease severity. Moreover, the AQLS significantly affects stigma.

## 1. Introduction

Acne vulgaris (AV) is a prevalent skin condition that affects up to 80% of individuals during adolescence. The most affected group is adolescents. Acne is a chronic inflammatory condition affecting the pilosebaceous unit and is characterized by seborrhea, comedones, papules, pustules, nodules, cysts, and, in some cases, scars and keloids. Acne commonly occurs on the face, which can exacerbate concerns about body image and social interactions. The psychological impact of AV is well documented, with associations with depression, anxiety, and low self-esteem. Research has consistently shown that individuals with acne experience higher levels of psychological distress compared to those without acne, affecting their overall quality of life [1,2,3].

Stigmatization plays a significant role in the lives of individuals with dermatological conditions like AV. Stigma refers to the negative beliefs, attitudes, and stereotypes that society directs toward specific groups or individuals based on certain characteristics. For those with visible acne, stigmatization can manifest as humiliation, devaluation, and discrimination, leading to internalized stigma. Internalized stigma, or self-stigma, occurs when individuals internalize these negative societal views or stereotypes, which can exacerbate psychological distress [4,5,6].

Stigmatization has been found to play a significant role in the lives of individuals with AV. Visibly noticeable AV has the potential to differentiate individuals from others, leading to stigmatization. Patients may develop fears about others’ reactions and face significant self-confidence issues due to misconceptions surrounding their condition [7,8]. Research has shown that patients with acne and associated scarring frequently experience stigmatization, which can lead to impaired quality of life, social withdrawal, and body image disturbances. These factors may further increase the risk of developing depression and social anxiety [9,10].

In this study, we aimed to evaluate the internalized stigma state of AV and its relationship with quality of life and disease severity. Additionally, we investigated the factors associated with internalized stigma.

## 2. Materials and Methods

### 2.1. Ethics

The Cumhuriyet University Ethics Committee approved our study (approval number: 2022-12/62, date: 14 December 2022). This cross-sectional observational study included 179 patients with AV who were followed up at our hospital between October 2022 and May 2023. We employed a convenience sampling strategy, which was chosen due to the practical challenges of recruiting a large and diverse sample within a limited time frame. While this approach may introduce selection bias, potentially affecting the generalizability of our findings, it allowed us to efficiently gather a sufficient sample size to explore the relationship between internalized stigma and AV. The study was conducted following the Declaration of Helsinki, the Patient Rights Act, and established ethical standards. Written informed consent was obtained from all participants, and they were thoroughly briefed about the study. The study excluded patients with co-occurring psychiatric disorders. By excluding these patients, we aimed to isolate the impact of AV on internalized stigma and quality of life, ensuring that our findings specifically reflect the experiences of those without additional psychological conditions that might independently influence the outcomes.

The sociodemographic characteristics of the patients (age, gender, marital status, education level, body mass index, marital status, smoking status, and alcohol consumption) and other parameters that may affect the quality of life (QoL) and internalized stigma were documented. Marital status was included as a proxy indicator of potential differences in social support systems, interpersonal stress, or psychosocial coping, which could plausibly affect levels of internalized stigma. Regarding education level, patients were categorized into three groups: primary education (including both primary and middle school), secondary education (high school), and higher education (university and postgraduate). This classification allows for the exploration of educational differences in health literacy and the perception of stigma. We also included smoking and alcohol use as exploratory variables, given their association with coping behaviors and psychosocial distress in the literature, particularly among adolescents and young adults. BMI was included as a continuous variable to explore potential associations between physical health status and internalized stigma.

In our study, we utilized the Dermatology Life Quality Index (DLQI), which is the most commonly used quality of life scale for dermatological conditions, the Acne Quality of Life Scale (AQLS), which is the most frequently used quality of life scale for acne patients, and the Internalized Stigma Scale (ISS), one of the key scales for assessing internalized stigma. The Global Acne Grading System (GAGS) was utilized to assess disease severity.

### 2.2. Internalized Stigma Scale

The Internalized Stigma Scale (ISS) was originally developed by Ritsher et al. to assess stigma in individuals with mental illnesses and has since been adapted for various other conditions beyond psychological disorders [11]. The Turkish version of the scale was validated, and its reliability was confirmed for use in mental health contexts by Ersoy et al. [12]. The ISS is a Likert-type scale consisting of 29 items that measure the internalization of stigma among patients. It encompasses five dimensions: stereotype endorsement (7 items), alienation (6 items), perceived discrimination (5 items), stigma resistance (5 items), and social withdrawal (6 items). The total ISS ranges from 4 to 91, with higher scores indicating more severe internalized stigmatization [11]. For our study, the ISS was chosen due to its comprehensive assessment of various facets of internalized stigma, which aligns with our objective to understand how stigma affects patients with AV.

While the ISS was originally designed for mental health conditions, its application to AV is based on its adaptability to different conditions involving visible attributes and social stigma. Adaptation for AV involved evaluating its relevance and content validity for patients experiencing stigma related to a dermatological condition. We utilized the ISS in this context to capture the nuanced ways in which internalized stigma manifests in individuals with AV.

### 2.3. The Dermatology Life Quality Index

The DLQI is the pioneering quality-of-life scale designed specifically for dermatological conditions. It consists of ten questions addressing six domains: symptoms and feelings, daily activities, leisure, work and school, personal relationships, and treatment. Each item is scored from 0 (not at all affected) to 3 (very much affected), with total scores ranging from 0 to 30. Higher scores reflect a greater impact of the disease on daily activities, employment, interpersonal relationships, leisure activities, and academic life. The validity of the Turkish version of the DLQI was established by Oztürkcan et al. [13].

### 2.4. Acne Quality of Life Scale

The AQLS, developed by Gupta et al., consists of nine items designed to evaluate the psychological impact of acne, particularly in relation to emotional distress, self-consciousness, and social interactions [14]. The Turkish version of the scale was validated by Demirçay et al. [15]. Each item is rated on a 4-point Likert scale ranging from 1 (“not at all”) to 4 (“very much”), reflecting the extent of suffering experienced by patients due to their acne. The total score, obtained by summing all responses, ranges from 9 to 36, with higher scores indicating greater impairment in quality of life [14].

### 2.5. Global Acne Grading System

The Global Acne Grading System (GAGS), developed by Doshi et al. [16], is a scoring system used to assess acne severity on the basis of the distribution and type of lesions on the body. Patients receive a global acne score ranging from 0 to 44. Acne severity is categorized via this score as follows: “No acne (0 points), mild (1–18 points), moderate (19–30 points), severe (31–38 points), and very severe (>39 points).” [16]. GAGS was chosen over other acne severity scales due to its well-established validity and ease of use in clinical settings, making it suitable for accurately assessing acne severity in our study population.

### 2.6. Statistical Analysis

We entered the collected data from this study into the SPSS software package (Version 22.0) for evaluation. We assessed the normality of the continuous data distribution using the Kolmogorov-Smirnov test. Descriptive statistics for quantitative variables were presented as mean, SD, Min–Max, frequencies, and percentages. The reliability of the ISS and its subscales was assessed by calculating Cronbach’s alpha coefficients for internal consistency. Linear regression analysis was used to evaluate the risk predictors for ISS. In the linear regression model, both continuous and categorical variables were included as independent variables. Categorical variables such as gender, marital status, and education level were dummy coded prior to analysis. Gender was coded as 0 = male, 1 = female; marital status as 0 = married, 1 = single; education level as 0 = primary and secondary education, 1 = higher education; smoking status as 0 = yes, 1 = no; drinking status as 0 = yes, 1 = no. This approach is supported in the statistical literature, and it allows categorical predictors to be interpreted alongside continuous variables in linear regression models. The Pearson correlation test was used to determine whether there was a statistically significant relationship between quantitative variables. *p* values less than *0.05* indicated statistical significance.

## 3. Results

### 3.1. Demographic and Clinical Characteristics

The sociodemographic and clinical features of the patients with AV are presented in Table 1. The mean age of the patients was 18.35 ± 4.11 years. Among the patients, 74.9% were female and 25.1% were male. Most patients were single (94.4%), with a small percentage married (5.6%). Regarding education, 5.6% had primary education, 32.4% had secondary education, and 62% had higher education. The mean body mass index (BMI) was 21.89 ± 3.19. The majority of patients did not smoke (86.6%) or drink alcohol (97.8%).

### 3.2. Quality of Life and Internalized Stigma Scores

The mean AQLS score of patients with AV was 11.03 ± 3.26, the mean DLQI score was 6.90 ± 5.33, the mean ISS score was 55.96 ± 14.55, and the mean GAGS score was 21.59 ± 6.58.

The Cronbach’s alpha coefficient for the entire ISS scale was calculated as 0.79. The subscales of the ISS showed the following alpha values: alienation (0.71), stereotype endorsement (0.74), perceived discrimination (0.75), social withdrawal (0.71), and stigma resistance (0.82).

### 3.3. Correlations

The ISS was significantly positively correlated with the AQLS score (*r* = 0.653, *p* < 0.001) and the DLQI score (*r* = 0.487, *p* < 0.001). The correlation between ISS and GAGS was weaker but still significant (*r* = 0.257, *p* = 0.006). The practical significance of the *0.257* correlation between GAGS and ISS suggests a moderate association where higher acne severity is associated with greater internalized stigma, though it is less pronounced compared to other correlations. The ISS subscales were found to have a significant correlation with AQLS and DLQI (Table 2).

### 3.4. Predictors of Internalized Stigma

Linear regression analysis was performed to predict the ISS variable by using the AQLS, DLQI, GAGS score, smoking, drinking, education level, marital status, body mass index (BMI), age, and gender variables. Accordingly, the AQLS positively and significantly predicts ISS (*β* = 0.521, *p* < 0.001). The DLQI score, GAGS score, smoking status, drinking status, education level, marital status, BMI, age, and gender did not significantly predict ISS in this model (*p* > 0.05). The regression model confirmed that the AQLS score was a positive predictor of ISS (Table 3).

## 4. Discussion

Dermatological patients are one of the groups most affected by stigmatization. In our study, the mean total ISS scores for patients with AV were notably high. The skin, as the most visible part of the body, plays a crucial role in interactions with the external world. AV, which affects prominent areas such as the face, can lead to significant distress and psychological impact when it is located on visible body parts. This likely contributes to the elevated ISS scores observed in these patients.

Schuster et al. reported that the level of internalized stigma is high in individuals with acne-induced hyperpigmentation [9]. Internalized stigma is common among acne patients, leading to feelings of shame and social withdrawal, which adversely affect their quality of life and health perceptions. Nalbant et al. reported that patients with AV and rosacea experienced high IS, which was significantly associated with a lower quality of life [17]. Similarly, another study revealed that patients with acne vulgaris and psoriasis vulgaris had comparable and higher total internalized stigma scores [6].

The stigma associated with acne is a significant concern, as individuals with appearance-altering conditions such as AV often experience internalized stigma and feelings of stigmatization [17,18]. Social media use, particularly on photo-centric platforms such as Facebook, can intensify these feelings through upward appearance comparisons, leading to heightened stigmatization in those with acne [19]. The internalization of negative attitudes and societal stereotypes related to acne has been shown to contribute to internalized stigma, adversely affecting the quality of life, perceived health, body image, and even depression levels in AV patients [18].

Stigma is significantly related to the severity of the disease. The ISS was significantly correlated with the GAGS score in this study. Previous studies have reported that there is a positive correlation between acne severity and ISS [7,18]. Stigmatization significantly impacts the well-being of individuals with acne, affecting their health-related quality of life, psychological distress, and the manifestations of somatic symptoms. Stigmatization can negatively impact individuals’ social, emotional, and psychological well-being, leading to social exclusion, feelings of worthlessness, and difficulties in interpersonal relationships. This may lead to a decrease in individuals’ quality of life [1]. In this study, it was found that the ISS and its subscales showed significant correlations with both the AQLS and the DLQI. Additionally, the mean AQLS and DLQI scores for patients with AV were elevated. Bilgiç et al. reported higher AQLS and DLQI scores in AV patients and reported a significant correlation between the ISS and both the AQLS and DLQI, which is consistent with the findings of our study [7]. Similarly, another study reported a significant positive correlation between the mean values of ISS and the AQLS [18].

Stigma has a profound impact on both quality of life and disease severity through several interconnected mechanisms. Research shows that individuals subjected to stigma often experience elevated levels of psychological distress, which can aggravate disease symptoms and result in poorer health outcomes. This distress is frequently associated with social isolation and diminished access to healthcare resources, further compromising the quality of life [20]. Additionally, stigma can lead to the internalization of negative self-perceptions, which may deter individuals from seeking essential medical care, thereby exacerbating disease severity [21].

Studies have demonstrated that individuals with acne frequently experience internalized stigma, which can lead to feelings of shame and worthlessness. Perceived stigma has also been shown to be a strong predictor of health-related quality of life, psychological distress, and somatic symptoms, significantly influencing overall well-being in those with acne [1]. Although alexithymia is not a predisposing factor for acne, it has been associated with impaired quality of life and increased stigmatization in acne patients, highlighting the complex interaction between psychological factors and the lived experience of acne [22]. Furthermore, adults with acne who feel stigmatized are at a higher risk for poor quality of life, with perceived stigma being the strongest predictor of acne-related quality of life, illustrating the detrimental effects of societal attitudes on the well-being of these individuals [23].

Quality of life can also influence stigma. Individuals with a higher quality of life may have better social support systems, stronger coping mechanisms, and more resources, which can mitigate the effects of stigmatization and help them manage stigma more effectively. Conversely, individuals with lower quality of life may experience more pronounced effects of stigma, potentially worsening their social, emotional, and psychological well-being. Thus, the relationship between quality of life and stigma is bidirectional, with each potentially impacting the other. Similarly, in our study, a bidirectional relationship was found between quality of life and stigmatization. Stigma is significantly related to low quality of life. The AQLS positively and significantly predicts ISS. Additionally, although only the AQLS emerged as a statistically significant predictor of internalized stigma (ISS) in our regression model, this does not necessarily indicate that the DLQI or GAGS are not relevant. Our correlation analysis showed moderate to strong associations between these variables. Given this, multicollinearity may have influenced the regression results. AQLS and DLQI, in particular, share conceptual and statistical overlap, which may have led to inflated standard errors and the masking of individual effects. To explore this further, we conducted a post-hoc regression excluding AQLS. In this model, DLQI became a statistically significant predictor of ISS. This suggests that shared variance among these interrelated constructs may have caused the explained variance to be attributed primarily to AQLS in the full model. Thus, while AQLS appears as the strongest predictor, interpretations of its isolated effect should be made cautiously, acknowledging the interconnected nature of these psychosocial variables. Similarly, Kotekoglu et al. identified negative quality of life as a significant and independent predictor of elevated internalized stigma [18]. Therefore, addressing stigmatization is essential, as it can persist even after effective medical treatment. This underscores the importance of early interventions and evidence-based approaches that integrate dermatology, psychology, social sciences, and policy-making [24]. Future research is needed to further explore the significance of stigma in dermatological diseases and to develop effective strategies for its prevention.

Our study has several limitations, including its single-center design and limited sample size. Future studies should involve larger, multicenter cohorts to improve the generalizability and reliability of the results. The single-center limitation affects the generalizability of the study findings, while the sample size influences the study’s power, precision, and reliability. Addressing these factors is crucial for interpreting the results and assessing their applicability to broader contexts. Additionally, the study did not include a control group. The absence of a control group is a critical limitation that affects the validity and generalizability of our findings. Additionally, while we identified several predictors of internalized stigma, the non-significant findings in our regression analysis suggest complexities that warrant further exploration. These complexities may reflect limitations in the model or unmeasured variables influencing stigma. The study fails to consider many other potential causes of stigma (societal norms and cultural beliefs, social inequality, lack of education, or personal experience or trauma). Future studies may include these variables to provide a more comprehensive analysis of factors contributing to stigma in AV patients. Future research should focus on exploring the nuances of stigma in dermatological diseases and developing effective strategies for its prevention. Further studies with diverse populations and control groups are needed to validate and expand upon these findings.

## 5. Conclusions

The results of the present study indicate that patients with AV often experience high levels of stigma. The findings suggest that internalized stigma is strongly associated with reduced quality of life and increased disease severity. The findings underscore the strong association between internalized stigma and reduced quality of life, with the Acne Quality of Life Scale emerging as a significant predictor of stigma levels. Given these insights, it is crucial for clinicians to integrate a comprehensive approach to managing AV that includes addressing internalized stigma. Clinicians should routinely assess internalized stigma as part of the standard evaluation for adolescents with acne vulgaris. Incorporating strategies to mitigate stigma, such as providing psychological support, counseling, and stigma-reduction interventions, should become a core component of acne treatment protocols.

The study calls for an urgent shift toward a holistic approach in acne management that not only targets the clinical aspects of the condition but also prioritizes the psychological well-being of patients. By addressing the psychological burden and stigma associated with AV, clinicians can improve patient outcomes and enhance overall disease management. This integrated approach will contribute to a better quality of life for patients and promote more effective and compassionate care.

## Figures and Tables

**Table 1:** Sociodemographic and clinical characteristics of acne vulgaris patients and comparison of internalized stigma scores.

Variables	n (%) or Mean ± SD (Min–Max) (n = 179)	Median ISS	*p*-Value
Age	20.35 ± 4.11 (13–37)		
Gender			0.305
Female	134 (74.9%)	56.0	
Male	45 (25.1%)	49.0	
Marital status			0.427
Single	169 (94.4%)	53.5	
Married	10 (5.6%)	62.0	
Education level			0.092
Primary education	10 (5.6%)	54.0	
Secondary education	58 (32.4%)	59.0	
Higher education	111 (62%)	52.5	
BMI, kg/m^2^	21.89 ± 3.19 (16–32)		
Smoking status			0.343
No	155 (86.6%)	54.0	
Yes	24 (13.4%)	52.0	
Drinking status			0.137
No	175 (97.8%)	54.5	
Yes	4 (2.2%)	42.0	
GAGS score	21.59 (6.58), (3–44)		
AQLS	11.03 (3.26), (1–19)		
ISS	55.96 (14.55), (29–109)		
DLQI	6.90 (5.33), (0–25)		

Abbreviations: BMI, body mass index; GAGS, Global Acne Grading System; AQLS, Acne Quality of Life Scale; ISS, Internalized Stigma Scale; DLQI, Dermatology Life Quality Index.

**Table 2:** Correlations between the Internalized Stigma Scale, the Acne Quality of Life Scale, the Dermatology Life Quality Index, and disease severity.

The ISS Subscales	The AQLS Correlation Coefficient *p*	The DLQI Correlation Coefficient *p*	GAGS Score Correlation Coefficient *p*
Alienation	0.636 <0.001	0.466 <0.001	0.145 0.109
Stereotype endorsement	0.448 <0.001	0.408 <0.001	0.207 0.022
Perceived discrimination	0.583 <0.001	0.466 <0.001	0.180 0.047
Social withdrawal	0.664 <0.001	0.482 <0.001	0.201 0.027
Stigma resistance	−0.176 0.055	−0.187 0.043	0.022 0.814
Total	0.653 <0.001	0.487 <0.001	0.257 0.006

Abbreviations: AQLS, Acne Quality of Life Scale; ISS, Internalized Stigma Scale; DLQI, Dermatology Life Quality Index; GAGS, Global Acne Grading System.

**Table 3:** The results of the statistical analysis of factors associated with internalized stigma in acne vulgaris patients.

Model	*β*	*p*	95% Confidence Interval	
			Lower Bound	Upper Bound
Constant	0.83	0.259	−0.627	2.287
AQLS	0.521	<0.001	0.200	0.526
DLQI	−0.159	0.228	−0.263	0.064
GAGS score	0.068	0.511	−0.010	0.019
Smoking status	−0.066	0.657	−0.436	0.277
Drinking status	0.160	0.197	−0.176	0.839
Education level	−0.42	0.804	−0.071	0.056
Marital status	0.097	0.597	−0.200	0.345
BMI, kg/m^2^	−0.022	0.830	−0.168	0.135
Age	−0.028	0.856	−0.041	0.034
Gender	0.150	0.165	−0.063	0.363

Abbreviations: AQLS, Acne Quality of Life Scale; DLQI, Dermatology Life Quality Index; GAGS, Global Acne Grading System; BMI, body mass index.

## Data Availability

The data presented in this study are available on request from the corresponding author.

## Abbreviations

The following abbreviations are used in this manuscript:

AQLS	Acne Quality of Life Scale
AV	Acne vulgaris
BMI	Body Mass Index
DLQI	Dermatology Life Quality Index
GAGS	Global Acne Grading System
ISS	Internalized Stigma Scale
